# Type and Timing of Negative Life Events Are Associated with Adolescent Depression

**DOI:** 10.3389/fpsyt.2018.00041

**Published:** 2018-02-14

**Authors:** Saori Nishikawa, Takashi X. Fujisawa, Masahiko Kojima, Akemi Tomoda

**Affiliations:** ^1^Research Center for Child Mental Development, Fukui University, Fukui, Japan

**Keywords:** negative life event, trait-resilience, posttraumatic stress symptoms, posttraumatic growth, adolescence

## Abstract

Previous studies have demonstrated an association between negative life events (NLEs) in childhood and resilience/posttraumatic growth (PTG) with regard to the pathogenesis of major depressive disorder. We hypothesized that the type and timing of NLEs interact to influence mental health in the general youth population. Therefore, the present study aimed to examine the effects of NLE timing and intensity on current depressive symptoms, and to determine the direct and indirect effects of NLEs/resilience on PTG and depression among non-clinical adolescents. Data were collected from 1,038 high-school students across seven high schools in Fukui, Japan, during their freshman and sophomore years (648 boys and 390 girls, mean age = 15.71, SD = 0.524). Respondents completed a set of questionnaires designed to evaluate the type and timing of NLEs, depressive and traumatic symptoms, and PTG. Cluster analysis was used to divide participants into three groups based on outcomes: “cluster 1” (*n* = 631), for whom depressive scores were significantly lower than other two subgroups (*p* < 0.05, for both); “cluster 2” (*n* = 52), for whom levels of current and past perceived stress associated with NLEs were significantly higher than those of the other two subgroups (*p* < 0.05, for both); “cluster 3” (*n* = 374), for whom perceived stress at the time of NLE was significantly higher than that of participants in the cluster 1 (*p* < 0.05) group, but not the cluster 2 group. Our findings indicated that exposure to NLEs at a younger age resulted in stronger negative outcomes and that NLE timing and intensity were associated with PTG and current symptoms of depression. Furthermore, path analysis demonstrated that associations between perceived stress at the time of NLEs were direct and indirect predictors of current depression *via* PTG and that posttraumatic stress symptom and PTG mediate the association between NLEs/trait-resiliency and current depression. In conclusion, our findings suggest that event intensity, NLE timing, and gender may play a role in emotional vulnerability during adolescence.

## Introduction

Most individuals have been exposed to some level of trauma during childhood. Previous research has indicated that negative life events (NLEs) such as bullying, traffic accidents, natural disasters, interpersonal problems, conflicts with parents, and poor academic performance are among the most robust predictors of poor well-being in adolescence (1–4). Additional research has suggested that middle school students experience and respond to NLEs differently than older students and that such differences contribute to heightened emotional vulnerability and more frequent emotional reactions (5).

Several studies have also reported that NLEs during childhood are significant predictors of psychiatric disorders in adulthood (6, 7) and that specific NLEs are associated with emotional problems (6, 8). For example, health-threatening events and stressful interpersonal relationships have been linked to depressive symptoms (6). Moreover, adolescents tend to be more vulnerable to depression, anger, and anxiety associated with NLEs (5). Exposure to traumatic events has also been identified as a risk factor for posttraumatic stress disorder (PTSD) and anxiety (9). Complex trauma is regarded as both exposure to multiple and/or chronic interpersonal traumatic experiences typically occurring within the caregiving system and the immediate and ongoing impact of this exposure across areas of development and functioning (10). Studies have revealed that individuals exposed to trauma during middle childhood report higher levels of depressive and PTSD symptoms in adulthood, relative to non-exposed individuals (11). Teicher and Samson (12) proposed the term “ecophenotype” to distinguish psychiatric conditions (e.g., major depressive disorder, anxiety, PTSD) based on the timing, type, and severity of exposure to maltreatment. Accumulating evidence suggests that emotional dysregulation plays a mediating role in the association between exposure to violence and posttraumatic stress symptoms (PTSS) in clinically referred adolescents (13).

However, not all adolescents exposed to NLEs experience poor mental health outcomes. In fact, previous studies have suggested that some individuals experience positive outcomes (14, 15) following NLE (e.g., breaking away from negative influences, triumphing over adversity, etc.). Tedeschi and Calhoun (15) defined posttraumatic growth (PTG) as “a phenomenon of a personal development as positive change in a person’s life as a result of highly traumatic events.” Early research on the relationship between NLEs and PTG demonstrated an association between fearfulness relating to the degree of stress and high levels of PTG (16). A more recent meta-analysis reported that this relationship approximates a “U-shaped” curve, and that PTG is similarly correlated with PTSS (17). That is, the association between PTG and PTSS intensity is such that very high or very low levels of stress are more likely to result in PTG (18). Additional studies have indicated that PTG leads to the development of resilience, indirectly inhibiting depressive symptoms and PTSS associated with additional NLEs (19, 20). Other studies have reported that PTG reduces later symptoms stress (21) but not those associated with depression (22).

Trait-resilience (ego-resiliency) is defined as the ability to adjust to environmental stress and change (23, 24). Trait-resilience is stable across childhood (25) and into adulthood (26), and previous studies have indicated that interactions between genes and environment play a role in regulating trait resilience. Taylor et al. (27) demonstrated that parenting style and genetic variations in serotonin levels are significant predictors of trait-resilience during early childhood. Individuals with high trait-resilience exhibit higher levels of adaptive flexibility during recovery from traumatic experiences (25). In addition, trait-resilience is positively associated with virtues (i.e., conscientiousness, vitality, and relationship) and PTG, while PTSD symptoms are not (28). One clinical study demonstrated that, while neuroticism and extraversion mediate the association between positive life events and resilience, NLEs directly contribute to trait-resilience (29). Resilience has also been associated with NLEs and mental health problems (30), acting as a mediating factor in the association between childhood maltreatment and psychiatric symptoms later in life (31).

**Figure 1:**
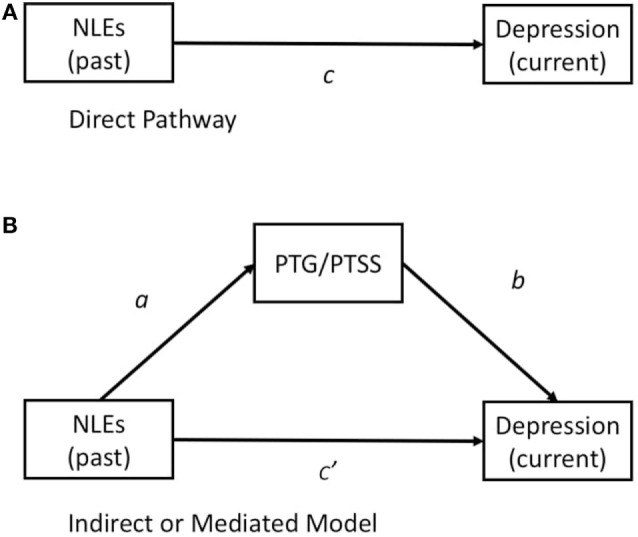
**(A)** Illustration of the direct effect of NLEs (past stress) on current depressive symptoms. **(B)** Illustration of a mediation model in which NLEs (past) are hypothesized to exert direct and indirect effects on depression *via* PTG or PTSS. Abbreviations: NLE, negative life event; PTG, posttraumatic growth; PTSS, posttraumatic stress symptoms.

Despite such findings, little is known regarding the risk and protective factors that characterize resilience and PTG among Japanese adolescents. Early research regarding PTSS focused on negative/stressful life events that are likely to cause narrowly defined trauma (e.g., wars and disasters), while few studies have focused on PTSS associated with NLEs in daily life. Thus, it is necessary to elucidate the effects of PTG and resilience on PTSS associated with NLEs. The present study aimed to examine the effects of NLE timing and intensity on current depressive symptoms and to determine the direct and indirect effects of NLEs/resilience on PTG and depression among non-clinical adolescents in Japan. We hypothesized (a) that NLE timing and stress intensity are associated with the development of PTG and current depressive symptoms, (b) that NLEs exert both direct and indirect effects on PTG and depression (see Figures 1A,B), and (c) that PTSS and PTG mediate the association between stress and current depressive outcomes (e.g., depression, stress related to NLEs, see Figure 1B).

## Materials and Methods

### Participants

The present study included 1,055 high-school students from Fukui prefecture (778,550 inhabitants). As 17 students were absent at the time of the study, a final total of 1,038 students (mean age = 15.71, SD = 0.524, range = 15–17 years; boys = 648, girls = 390) were included (see Table 1).

**Table 1:** Demographic characteristics (age and gender) of the participants.

	Age				*t*	*p*-Value
	Mean	SD	Minimum	Maximum		
Boys (*n* = 648)	15.68	0.51	15	18	2.50	0.013
Girls (*n* = 390)	15.77	0.55	15	18		
Total (*n* = 1,038)	15.71	0.52	15	18	–	–

### Procedure

The study protocol was approved by the ethical committee at the University of Fukui (approval number: 834). The purposes of the present study were announced at a meeting of high-school principals from Fukui Prefecture, at which time their assistance and cooperation were requested. Principals and individual classroom teachers provided consent to include 29 classes from seven high schools. A researcher and technician visited schools and conducted the study. The purposes and voluntary nature of the present study were explained to parents by a letter and to students orally and in writing, following which they were requested to submit a letter of consent if they wished to participate in the study. Participating students completed the questionnaires anonymously during regular class hours. Identifying information included only gender, age and class. Students took approximately 30 min to complete the questionnaires.

### Measures

#### Type and Timing of NLEs

Participants were asked to select the types of stressful or traumatic life events they had experienced from among a list of the following 10 options: death of family member/close friend, parental divorce, conflict with parents, academic failure, domestic economic issues, bullying, interpersonal conflicts, serious illness or injury, accident or disaster, and other. Participants were then asked to rate how much stress they had experienced at the time of the NLE, as well as their current level of stress associated with the NLE, on a Likert scale ranging from 1 (low stress) to 4 (high stress).

#### Posttraumatic Stress Symptom

Following the NLE questionnaire, participants completed the Impact of Event Scale-Revised (IES-R), which includes 22 items across three subscales designed to assess symptoms of posttraumatic stress: intrusion, avoidance, and hyperarousal in the past 7 days (32). Each item was rated on a five-point Likert scale, ranging from 0 (not at all) to 4 (a very great degree). Higher scores indicate higher levels of traumatic symptoms. The Japanese version of the IES-R has demonstrated good validity and test–retest reliability in previous studies (33).

#### Posttraumatic Growth

The Japanese version of the Posttraumatic Growth Inventory (PTGI-J) (34) was developed based on the original version of the PTGI (35), which consists of 21 items designed to assess levels of PTG in individuals who have experienced traumatic events. Participants were asked to provide responses on a 6-point Likert scale ranging from 0 (*I did not experience this change as a result of my crisis*) to 5 = (*I experienced this change to a very great degree as a result of my crisis*).

#### Depression

The severity of depressive symptoms was evaluated using the Birleson Depression Self-Rating Scale for Children (DSRS-C) (36), which consists of 18 items designed to assess symptoms of depression within the past week. Responses were provided on a Likert scale ranging from 0 (never) to 2 (mostly). The validity and reliability of the Japanese version of the DSRS-C have been confirmed in a previous study (37).

#### Trait Resilience

The Ego-Resiliency Scale (ER-89) (38) is a unidimensional, 14-item self-report scale designed to measure trait resilience (i.e., ego-resiliency) in adolescents. Participants provided responses on a 4-point Likert scale, ranging from 1 (*does not apply at all*) to 4 (*applies very strongly*). The Japanese version of the ER-89 has demonstrated good reliability and validity in previous studies (39).

### Variables and Statistical Methods

The Statistical Package for Social Sciences (SPSS) version 22 (IBM, 2013) was used for performing descriptive statistics, correlations, and analyses of variance (ANOVAs). Path models developed using Structural Equation Models on AMOS 22 were used to evaluate the relationships among variables. To investigate whether a variable (PTG) is a mediator between independent variable (*NLEs past*) and dependent variable (*depression current*) in the path analysis, we first drew a direct path from *NLEs past* to *depression current* (Figure 1A). In the next analysis, two paths were added (Figure 1B): one from *NLEs past* to *PTG* and another from *PTG* to *depression current*. If *PTG* is a significant mediator, the weight of the path from *NLEs past* to *depression current* will decrease in the second analysis, relative to that in the first analysis (40). The goodness of fit index (GFI) is considered a reasonable statistical index for evaluating a model and was used to assess the fit between a hypothesized model and the data. AMOS was used to evaluate the following parameters of each model based on different theoretical perspectives: CMIN/df (the minimum value of sample discrepancy divided by its degree of freedom), in which smaller values are preferable (41), the comparative fit index (CFI), a measure of the relative amount of variance and covariance (values over 0.9 are preferable) (42), the root mean square error of approximation (RMSEA) based on population discrepancy (values below 0.08 are preferable) (43), and the incremental fit index (IFI) (values over 0.9 are preferable) (44).

## Results

### Descriptive Statistics

The distributions of variables (ER-89, PTSS, depression) were approximately bell-shaped, with the exception of those for past NLEs (stress intensity at the time of NLE), current NLEs (current stress intensity associated with the NLE), and PTSS (which exhibited peaks at the low end). Table 2 shows the means (SDs, skewness, kurtosis) and correlations between PTSS, DSRS-C scores, ER-89 scores, and other variables. PTSS was positively correlated with DSRS-C scores, PTG, past NLEs, current NLEs (*r* = between −0.17 and −0.23, *p* < 0.001 and 0.05), and negatively correlated with ER-89 scores (*r* = −0.10, *p* = 0.001). PTG was positively correlated with ER-89 scores and past NLEs (*r* = between −0.17 and −0.23, *p* < 0.001 and 0.05), and negatively correlated with DSRS-C scores and current NLEs (*r* = between −0.17 and −0.23, *p* < 0.001 and 0.05). Gender (1 = boy, 2 = girl) was positively correlated with PTSS, DSRS-C scores, past NLEs, and current NLEs. Further correlational analysis was conducted separately based on gender. Trait-resilience and PTSS were negatively correlated among girls only (*r* = −0.15, *p* = 0.004), while PTG and PTSS were positively correlated among boys only (*r* = 0.17, *p* = 0.000). One-way ANOVA indicated that girls scored significantly higher on the DSRS-C (*p* = 0.01) and had significantly higher levels of PTSS (*p* = 0.000), stress intensity at the time of NLEs (*p* = 0.000), and current stress associated with NLEs (*p* = 0.021) than boys.

**Table 2:** Means, SDs, and correlations among study variables.

	Mean (SD)	Skewness	Kurtosis	1	2	3	4	5	6	7
1. Gender (1 = boys, 2 = girls)	1.38 (0.49)	0.514	−1.739	–						
2. Posttraumatic stress syndrome	21.15 (17.48)	0.748	−0.098	0.141**	–					
3. Depressive symptoms	11.61 (5.87)	0.443	−0.022	0.078*	0.440**	–				
4. Trait resilience	21.18 (7.84)	0.049	−0.317	−0.041	−0.099**	−0.572**	–			
5. PTG	44.71 (22.24)	−0.086	−0.670	0.016	0.136**	−0.376**	0.516**	–		
6. Past NLEs (stress)	2.82 (0.98)	−0.295	−0.987	0.177**	0.360**	0.226**	−0.001	0.136**	–	
7. Current NLEs (stress)	1.68 (0.82)	1.146	0.761	0.071*	0.375**	0.391**	−0.162**	−108**	0.368**	–

*p < 0.05, **p < 0.001. NLEs, negative life events; PTSS, posttraumatic stress symptoms; PTG, posttraumatic growth; Past NLEs, stress intensity at the time of NLE, Current NLEs, current stress intensity associated with the NLE.

Table 3 lists the frequencies and percentages of NLEs, the timing of exposure (age group), and the mean values of the other variables. A univariate *F*-test revealed a significant main effect of NLE timing on depressive symptoms, PTSS, and PTG. *Post hoc* analysis indicated that individuals who experienced NLEs after high school (high-school group) reported significantly higher levels of depressive symptoms than those who experienced NLEs in junior high school (*p* < 0.05). The high-school group also exhibited significantly higher levels of PTSS than elementary school and junior high-school groups (*p* < 0.05 for both), indicating that more recent NLEs had a greater impact on outcomes. Furthermore, a univariate *F*-test excluding participants (*n* = 255) who reported more recent NTEs (high-school group) revealed a significant main effect of NLE timing on PTSS (*p* < 0.05 for both). *Post hoc* analysis indicated that individuals who experienced NLEs before elementary school (*≦*6 years old) reported significantly higher levels of PTSS than those who had experienced such events in elementary or junior high school (*p* < 0.05 for both).

**Table 3:** Mean scores (SDs) of adolescents in each age group based on the NLE timing.

	Age at time of NLEs			*F*	*p*-Value	*Post hoc* comparisons
	A. before elementary	B. elementary school	C. junior high school			
Depression	11.92 (6.47)	11.70 (6.02)	11.08 (5.72)	1.04	ns	ns
Trait-resilience	19.43 (8.12)	20.50 (8.13)	21.69 (7.52)	2.85	ns	ns
PTSS	27.32 (23.51)	19.60 (17.00)	21.07 (17.13)	3.20	0.027	A > B and C
PTG	38.32 (20.72)	42.97 (1.62)	46.35 (21.87)	3.63	0.041	A < B and C
Past NLEs	2.62 (1.26)	2.73 (1.02)	2.83 (0.94)	1.26	ns	ns
Current NLEs	1.89 (1.10)	1.46 (0.71)	1.60 (0.76)	5.39	0.005	A > B and C

*NLEs, negative life events; PTSS, posttraumatic stress symptoms; PTG, posttraumatic growth; Past NLEs, stress intensity at the time of NLE; Current NLEs, current stress intensity associated with the NLE*.

### Type and Timing of NLEs

**Figure 2:**
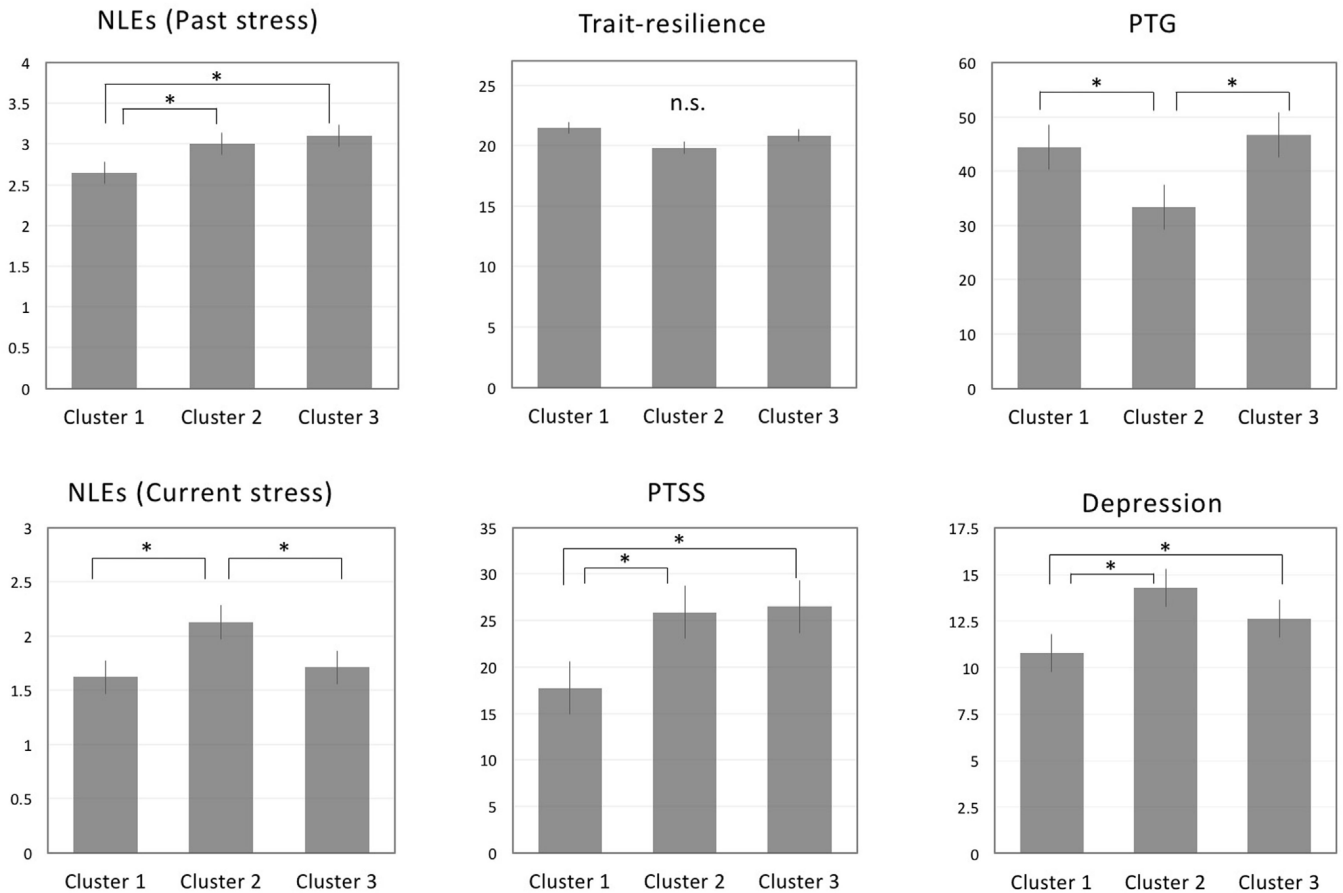
Comparisons regarding perceived stress at the time of NLE, trait-resilience, PTSS, and PTG across clustered NLEs. Note: *p < 0.05; abbreviations: NLE, negative life event; PTSS, posttraumatic stress symptoms; PTG, posttraumatic growth.

We used SPSS 22 to perform a cluster analysis of the sample of 1,038 adolescents in order to identify the subtypes of mental health variables (i.e., depression, PTSS, PTG, perceived past/current stress) in relation to NLE types. First, mean values of mental health variables were calculated for each NLE types. Clustering was performed using Ward’s linkage and agglomeration schedule. Three cluster solutions were identified into the following subgroups: cluster 1, cluster 2, and cluster 3. Participants in the first cluster (*n* = 631) reported significantly lower levels of depression than those in the second and third clusters (*p* < 0.05, for both). Participants of the second cluster (*n* = 52) reported significantly higher current and past NLEs associated with NLEs than those of the cluster 1 and cluster 2 (*p* < 0.05 for both). Participants of the third cluster (*n* = 374) reported experiencing more stress at the time of the NLE than those of the first cluster (*p* < 0.05), but not those of the second cluster. Finally, participants of both the cluster1 and cluster 3 groups reported higher levels of PTG than those of the cluster 2 (*p* < 0.05, for both). There were no significant differences in trait-resilience scores among the three groups (Figure 2). Table 4 shows the cluster characteristics identified by the pairwise comparisons, while Table 5 shows the results of *post hoc* analysis.

**Table 4:** Cluster groupings based on NLE type.

Types of negative life event	% (*n*)	Cluster
1. Death of family member/close friend	11.5 (119)	Cluster 1
2. Parental divorce	3.5 (36)	Cluster 3
3. Conflict with parents	5.0 (52)	Cluster 2
4. Academic failure	27.6 (286)	Cluster 1
5. Domestic economic issues	6.6 (69)	Cluster 1
6. Bullying	23.0 (239)	Cluster 3
7. Interpersonal conflicts	9.5 (99)	Cluster 3
8. Serious illness/injury	1.1 (11)	Cluster 1
9. Accident or disaster	12.2 (127)	Cluster 1

**Table 5:** Mean scores (SDs) of adolescents classified by cluster group.

	Group			*F*	*p*-Value	*Post hoc* comparisons
	Cluster 1	Cluster 2	Cluster 3			
Depression	10.79 (5.62)	14.31 (5.71)	12.65 (6.05)	17.74	0.000	Cluster 2 > Cluster 1, Cluster 3
Trait-resilience	21.48 (7.90)	19.83 (8.02)	20.85 (7.68)	1.55	ns	
PTSS	17.75 (16.07)	25.90 (18.29)	26.48 (18.29)	8.45	0.000	Cluster 2 > Cluster 1
PTG	44.50 (22.32)	33.35 (21.79)	46.75 (21.71)	32.14	0.000	Cluster 1, Cluster 2 > Cluster 3
Past NLEs	2.65 (0.99)	3.00 (0.97)	3.10 (0.88)	26.06	0.000	Cluster 2 > Cluster 1, Cluster 3
Current NLEs	1.62 (0.79)	2.13 (1.10)	1.71 (0.82)	9.88	0.000	Cluster 2 > Cluster 1, Cluster 3

*NLEs, negative life events; PTSS, posttraumatic stress symptoms; PTG, posttraumatic growth; Past NLEs, stress intensity at the time of NLE; Current NLEs, current stress intensity associated with the NLE*.

### Path Analysis

**Figure 3:**
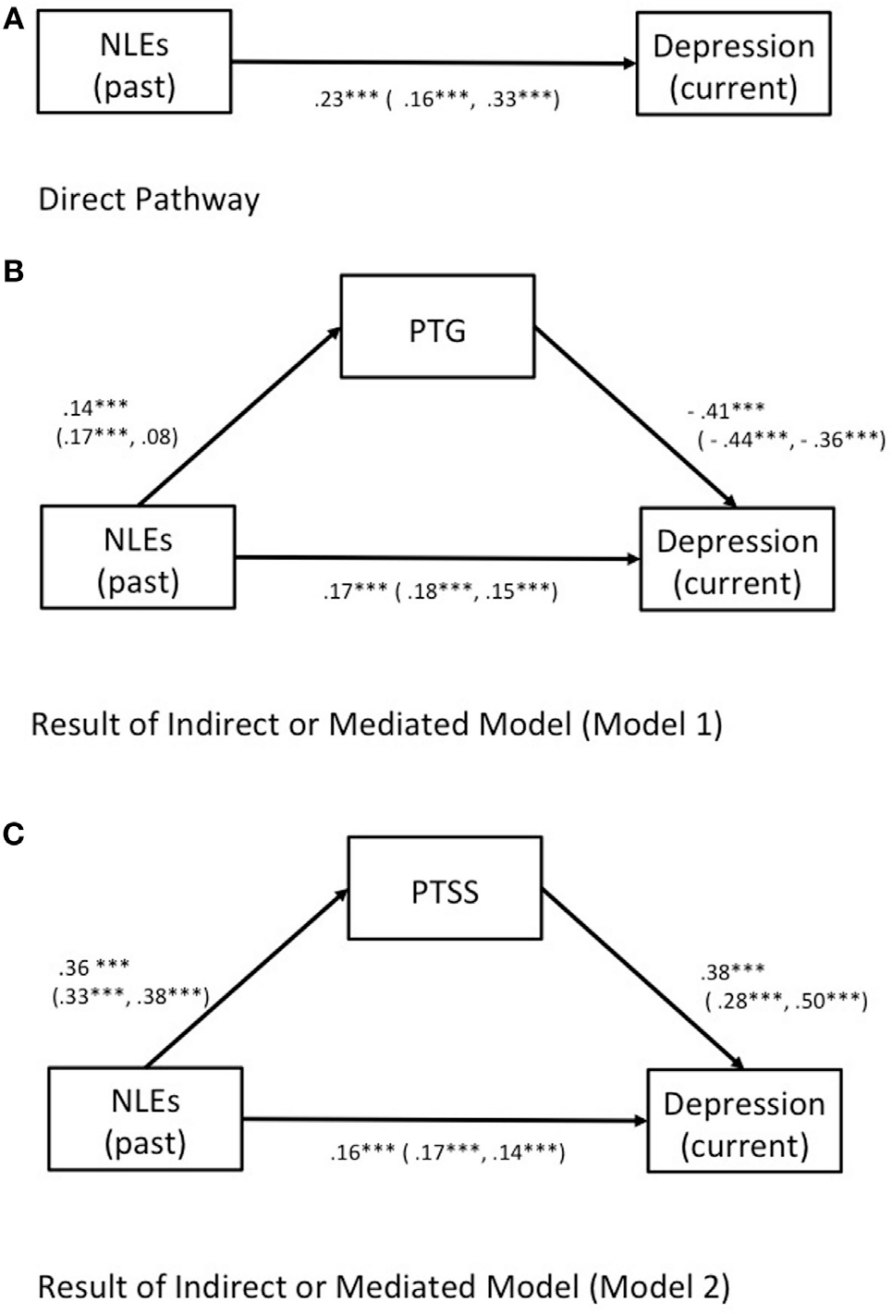
**(A)** Results of direct model in which negative life events (NLEs) (past stress) as a predictor of current depression. **(B)** Results of a mediation model in which NLEs (past stress) are hypothesized to exert direct and indirect effects on depression *via* posttraumatic growth (PTG). **(C)** Results of a mediation model in which NLEs (past stress) are hypothesized to exert direct and indirect effects on depression *via* PTSS. Note: standardized estimates of the direct and indirect effects on the depressive symptoms presented for all participants (male, female), respectively.

After the correlational analysis, the following strategy was used to examine the direct and indirect effects of each variable. The estimated model with standardized path coefficients is presented in Table 6. First, a direct-pathway model was constructed using past NLEs (stress at the time of NLEs) as a predictor of current depression. The pathway linking past NLEs and depression was significant (*p* < 0.001, see Figure 3A), with acceptable GFIs [*F* (1, 9) = 3.90, *p* = 0.048; CMIN/df = 3.895, CFI = 0.946, RMSEA = 0.530, IFI = 0.946].

**Table 6:** Fit indices of the models.

Model	*x*^2^ (df)	CMIN/df	CFI	RMSEA	IFI
Model 1 (All)	9.02 (1)	9.018	0.971	0.088	0.971
Boys	9.63 (1)	9.63	0.950	0.116	0.951
Girls	0.54 (1)	0.538	1.00	0.000	1.000
Model 2 (All)	8.80 (1)	8.803	0.979	0.087	0.979
Boys	10.61 (1)	10.609	0.936	0.122	0.938
Girls	0.04 (1)	0.042	1.000	0.000	1.005
Model 3 (All)	9.08 (1)	9.076	0.988	0.088	0.988
Boys	9.61 (1)	9.605	0.977	0.115	0.977
Girls	1.51 (1)	1.512	0.998	0.036	0.998
Model 4 (All)	2.34 (2)	2.388	1.00	0.014	1.000
Boys	1.47 (2)	0.733	1.000	0.000	1.001
Girls	3.73 (2)	1.864	0.997	0.047	0.998

*Note, CMIN/df, the minimum value of sample discrepancy divided by its degree of freedom*.

*CFI, comparative fit index; RMSEA, root mean square error of approximation; IFI, incremental fit index*.

Model 1 was developed in order to assess the direct and indirect effects of PTG as a mediator of depression (see Figure 3B). The pathways linking NLEs, PTG, and depression were all significant (*p* < 0.001 for all), indicating a partial mediation. All exhibited acceptable GFIs [*F* (1, 9) = 9.018, *p* = 0.003; CMIN/df = 9.018, CFI = 0.971, RMSEA = 0.088, IFI = 0.971, see Table 6]. All paths in Model 1 were significant across gender (*p* < 0.001). However, GFIs appeared to be non-acceptable for boys [*F* (1, 9) = 9.634, *p* = 0.002; CMIN/df = 9.634, CFI = 0.950, RMSEA = 0.116, IFI = 0.951], while a good fit was observed among girls [*F* (1, 9) = 0.538, *p* = 0.463; CMIN/df = 0.538, CFI = 1.000, RMSEA = 0.000, IFI = 1.004; see Table 6]. Model 2 was constructed to assess the direct and indirect effects of PTSS as a mediator of depression (see Figure 3C). The pathways linking NLEs, PTSS, and depression were all significant (*p* < 0.001 for all), and all exhibited acceptable GFIs [*F* (1, 9) = 8.80, *p* = 0.003; CMIN/df = 8.803, CFI = 0.929, RMSEA = 0.087, IFI = 0.979; see Table 6]. Good GFIs were observed among girls [*F* (1, 9) = 0.04, *p* = 0.837; CMIN/df = 0.042, CFI = 1.000, RMSEA = 0.000, IFI = 1.005], while the model did not fit the data very well among boys (see Table 6).

**Figure 4:**
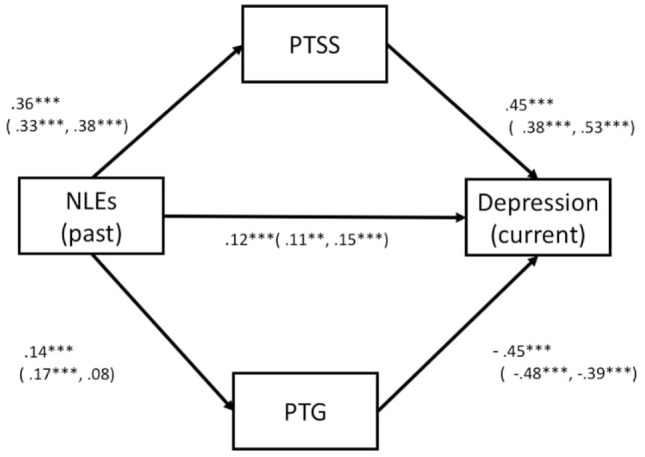
Multi and mediated model (Model 3). Illustration of a mediation model in which NLEs (past) is hypothesized to exert indirect effect on current depression associated with NLEs (past) *via* PTSS and PTG. Note: standardized estimates of the direct and indirect effects on depressive symptoms are presented for all participants (male, female), respectively. Abbreviations: NLE, negative life event; PTSS, posttraumatic stress symptoms; PTG, posttraumatic growth.

Model 3 (See Figure 4) was constructed with multiple mediators (i.e., PTSS, PTG), in which NLEs exhibited direct and indirect effects on depression (current) *via* PTSS and PTG. The pathways linking NLEs, PTG, PTSS, and depression were all significant (*p* < 0.001 for all), and all exhibited acceptable GFIs [*F* (1, 14) = 9.08, *p* = 0.003; CMIN/df = 9.076, CFI = 0.988, RMSEA = 0.088, IFI = 0.988, see Table 6].

**Figure 5:**
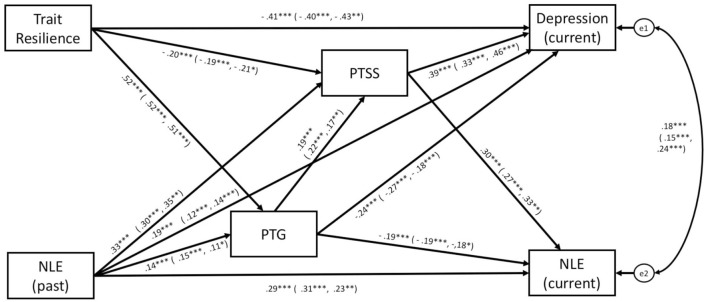
Multi and mediated model (Model 4). Illustration of a mediation model in which trait-resilience and past stress are hypothesized to exert indirect effects on current negative outcomes (current depression and stress associated with NLEs) *via* PTSS and PTG. Note: standardized estimates of the direct and indirect effects on depressive symptoms are presented for all participants (male, female), respectively. Abbreviations: NLE, negative life event; PTSS, posttraumatic stress symptoms; PTG, posttraumatic growth.

Finally, Model 4 (Figure 5) was constructed using predictors (NLEs past and trait-resilience) as well as outcome variables (depression and NLEs current) in order to assess the direct and indirect effects of any mediating variables (PTSS and PTG). The pathways linking NLEs past, PTG, trait-resilience, and depression were all significant (*p* < 0.001 and *p* < 0.005, see Figure 4), thus suggesting a partial mediation. The overall model fit the data well, *F* (2, 27) = 2.388, *p* = 0.303; CMIN/df = 1.19, CFI = 1.000, RMSEA = 0.000, IFI = 1.000 (see Table 6), indicating that PTSS and PTG mediate the effects of past NLEs and trait-resilience on current depressive symptoms and stress associated with NLEs. Additionally, we used a bootstrap examination method (with 5,000 bootstrap samples). If the CIs did not include 0, it could indicate that the mediating effects were statistically significant (45). In the present study, the mediating effects (expect a path model linking between trait-resilience and depression) were all significant. The results of bootstrap analysis indicated that the indirect effect of past stress on depression *via* PTG or PTSS were significantly different from 0 (see Data Sheet S1 in Supplementary Material).

The study conducted the analysis separately in gender. Except for the path linking past NLEs and PTG among girls, all paths in Model 3 were significant across gender (*p* < 0.001, see Figure 4). Among both boys and girls, all paths in Model 4 (see Figure 5) were significant (*p* < 0.001 and *p* < 0.05, see Table 6). GFIs appeared acceptable for boys [*F* (2, 27) = 1.466, *p* = 0.481, CMIN/df = 0.733, CFI = 1.000, RMSEA = 0.000, IFI = 1.001] and girls [*F* (2, 27) = 3.728, *p* = 0.155; CMIN/df = 1.864, CFI = 0.997, RMSEA = 0.047, IFI = 0.999; see Figure 5].

## Discussion

The primary aims of the present study were to examine the effects NLE timing and intensity on PTSS, PTG, and depressive symptoms; and to explore the direct and indirect effects of NLEs on PTG and depression among non-clinical adolescents in Japan. Our findings indicated that NLE timing and intensity were associated with PTG and current symptoms of depression. Furthermore, path analysis demonstrated that associations between perceived stress at the time of NLEs were direct and indirect predictors of current depression *via* PTG and that PTSS and PTG mediate the association between NLEs/trait-resiliency and current depression.

Although trait-resilience was not associated with perceived stress concerning a particular NLE in the present study, our findings indicate that this factor directly and indirectly influences current depression among adolescents. The present results are consistent with those of previous studies (30, 31), which have demonstrated associations among resilience, child maltreatment, and later depressive symptoms. Thus, trait-resilience may be regarded as an independent construct that relies on the level of PTG to indirectly improve symptoms of depression.

In accordance with the findings of early studies (6, 8), our findings indicated that different types of NLEs were associated with variations in PTG levels and outcomes (e.g., PTSS and depression).

Among the three cluster-groups of the present study, adolescents who reported conflict with parents (i.e., cluster 2) tended to have higher levels of PTSS and lower levels of PTG, while those of the cluster 1 or cluster 3 exhibited higher levels of PTG, resulting in lower levels of PTSS and current depression. Previous studies have indicated that relationships with parents influence well-being during childhood and adolescence (46). As adolescence is the period during which individuals begin to develop autonomy and independence (47), parent–child conflict is more likely to occur. However, in the present study, conflict with parents was the most robust predictor of poor outcomes such as current depression, high PTSS, and low PTG. These findings are consistent with those of a previous study, which demonstrated that parenting style had a direct effect on PTSS *via* resilience (48). We observed that depressive symptoms were less severe in students who had experienced trauma from cluster 1, indicating that some students may have been able to overcome such life events (e.g., death of family member, academic failure, etc.). Furthermore, the cluster 1 trauma group exhibited less severe depression than the cluster 2 group, in which conflicts with parents were noted, indicating that such conflicts may continue to affect the mental health of students. However, we did not investigate the types of parental conflicts with which students were struggling. As the severity of these conflicts may also differ among students, further studies are required to examine the influence of various types of parental conflict.

The results of the present study are consistent with those of Mann et al. (5), who reported that adolescents are more vulnerable than young adults to more recent NLEs even when they are relatively low in intensity. Our findings suggest that exposure to NLEs at a younger age resulted in stronger negative outcomes during adolescence, in accordance with the findings of Dunn et al. (11). Adolescents exposed to NLEs prior to reaching school age reported higher levels of PTSS and PTG than those who experienced NLEs in elementary and junior high school. These results suggest that individuals exposed to NLEs prior to the age of 6 are more emotionally vulnerable than non-exposed individuals during adolescence, even among the non-clinical population. However, these adolescents were more likely to experience PTG than those who experienced NLEs after entering elementary school, indicating that the developmental stage at which NLEs occur may predict the level of PTG (8). Moreover, time elapsed after the trauma may be associated with negative outcomes. Such adolescents may go through positive psychological change from childhood to adolescence.

In the present study, path analysis demonstrated an association between perceived stress at the time of the NLE and PTG. Individuals with stable trait-resilience reported greater PTG, which reduced PTSD symptoms and current depressive syndrome. Model 1 demonstrated a mediating relationship between stress intensity at the time of NLE, PTG, and depression. Greater PTG was associated with lower levels of current depressive symptoms. Model 2 was used to examine the mediating relationship between stress intensity at the time of NLE, PTSS, and depression. However, this model did not achieve critical GFI values. Model 3, which was constructed using multiple mediators (i.e., PTSS and PTG), indicated that NLEs directly influenced depression and PTSS, and that PTG mediated the association between NLEs and depression. Finally, Model 4 demonstrated the direct and indirect effects of perceived stress at the time of the NLE and trait-resilience, which were identified as predictors of current stress intensity (NLEs current) and depression. Our results indicated that these associations were mediated by PTSS and PTG.

Significant differences in some GFI values were observed across gender. For example, GFIs for Model 2 were acceptable among boys, while those for girls were slightly worse than the critical values. Although these findings suggest that gender plays a role in mediating these effects, the sample size may have influenced our results. Thus, further studies are required to clarify the effect of gender on these associations. The results of the present study are in accordance with those of a recent study, which indicated that adolescents who had been exposed to one or several traumatic experiences reported more internal and external problems than those who had internalizing and externalizing problems compared to those who had not been exposed to such experiences. The authors also demonstrated associations between cluster 2 and internal/external conflicts (49). Further studies are needed to examine whether current depressive symptoms might be a cause of (rather than an effect of) increased exposure to stressors and the degree of their negative affect.

The present study possesses some limitations of note. First, as our study utilized a cross-sectional design, we were unable to determine causal associations. The present study has struggled with the emerging consensus in the field regarding the issue of using cross-sectional data to test mediation (50). Second, there are several limitations to mention about the sample used in the present study. We were unable to account for the influence of socioeconomic status, as this information was not obtained in order to protect the privacy of the students. The unequal numbers of boys and girls is one of the major limitations. The present study was conducted at four general and three industrial high schools. The reason for the current gender distribution is that industrial high schools typically have a higher proportion of boys compared to girls. It is difficult to say that the youth of Fukui are representative of Japan unless more participants from other prefectures are included, and there has been, to date, no normative study of adolescent mental health problems conducted in Japan. Third, as the NLE questionnaire used in the present study included current NLEs, a group of students (*n* = 255) who reported recent NLEs were excluded in order to examine the effects of past NLEs on outcomes. Thus, we were unable to determine the full effects of past NLEs on outcomes for all participants. Based on Cook’s definition of complex trauma (10), it might be possible to interpret the cluster 1 as “single incident trauma,” cluster 2 as “complex trauma,” and cluster 3 as “combined trauma.” However, it is clear that these should be labeled with more scientific basis. Further studies are required to determine the effects of different types of trauma on mental health outcomes among non-clinical adolescents. Fourth, lack of the replicability of the cluster analysis (51) is another issue of the present study. The clusters failed to replicate across random subsamples even though similarities of patterns were observed across the groups (see Data Sheet S1 in Supplementary Material). It is necessary for the further studies to investigate by using alternative methods for providing internal validity. Finally, we did not examine the influence of other psychosocial factors such as social support (52), meaning-making (e.g., to improve adjustment to the stressful event) (53), and coping (54), and others (55) on PTG. Future studies should focus on such analyses in order to provide new directions for interventions following NLEs.

Despite these limitations, our results demonstrated an association between perceived stress associated with NLEs and current depressive symptoms, and that this association was mediated by PTSS and PTG in a large sample of Japanese adolescents. Moreover, our findings indicated that event intensity, NLE timing, and gender play a role in levels of emotional vulnerability during adolescence. Thus, clinicians and educators should consider the timing of NLE exposure (11) as well as the severity of NLEs in the promotion of social-cognitive skills (56) and trait-resilience.

## Ethics Statement

The study protocol was approved by the ethical committee at the University of Fukui (approval number: 834). The purposes of the present study were announced at a meeting of high-school principals from Fukui Prefecture, at which time their assistance and cooperation were requested. Principals and individual classroom teachers provided consent to include 29 classes from seven high schools. A researcher and technician visited schools and conducted the study. The purposes and voluntary nature of the present study were explained to parents by a letter and to students orally and in writing, following which they were requested to submit a letter of consent if they wished to participate in the study. Participating students completed the questionnaires anonymously during regular class hours. Identifying information included only gender, age, and class. Students took approximately 30 min to complete the questionnaires.

## Author Contributions

TF and MK were involved in recruiting the participants and conducting the experiment. SN and TF were involved in analyzing and interpreting data, and drafting the article. AT conceived of the study, participated in its design and coordination, and drafted the manuscript. All the authors have read and approved the final manuscript.

## Conflict of Interest Statement

The authors declare that the research was conducted in the absence of any commercial or financial relationships that could be construed as a potential conflict of interest.
