# Supplementary material for: Type and Timing of Negative Life Events Are Associated with Adolescent Depression
Source: Front Psychiatry. 2018 Feb 14;9:41. doi: 10.3389/fpsyt.2018.00041 (PMC5817059; doi:10.3389/fpsyt.2018.00041)
Supplement: Supplementary file 1 [file data_sheet_1.PDF]

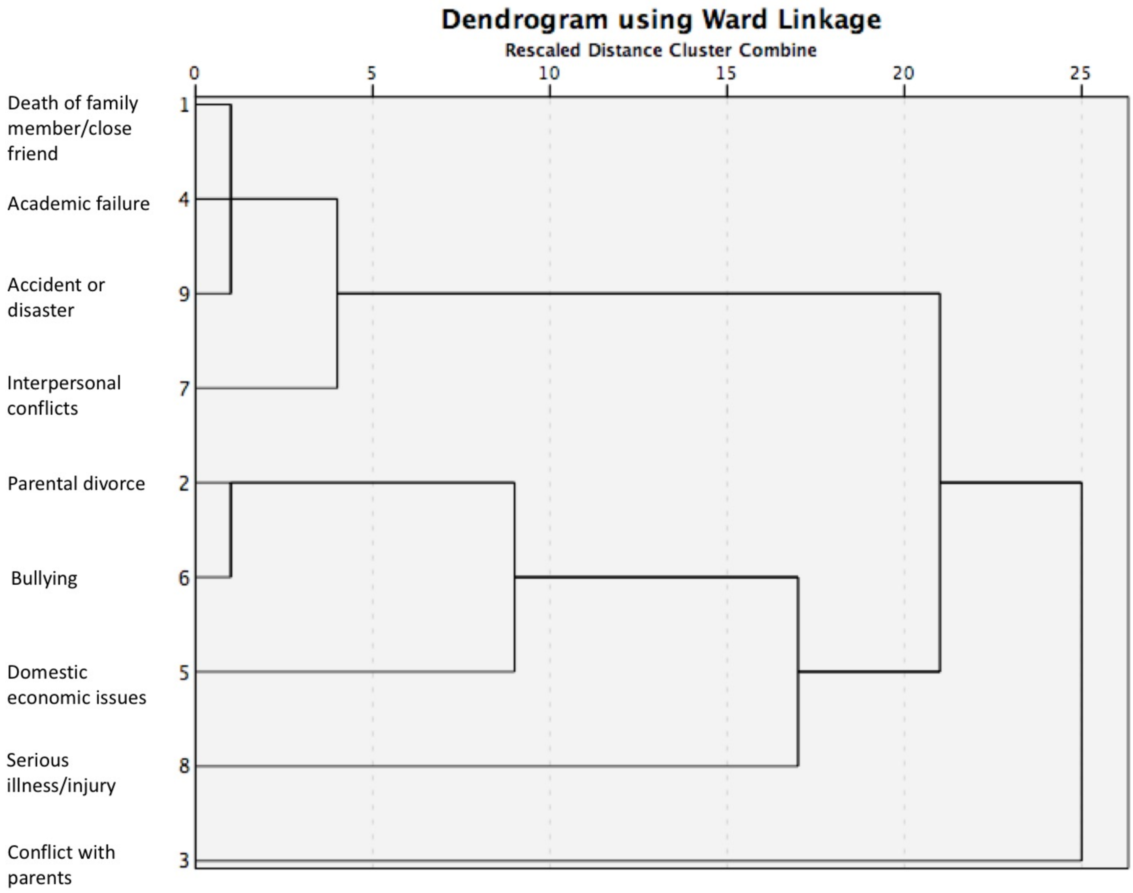

Note: group 1, n=51

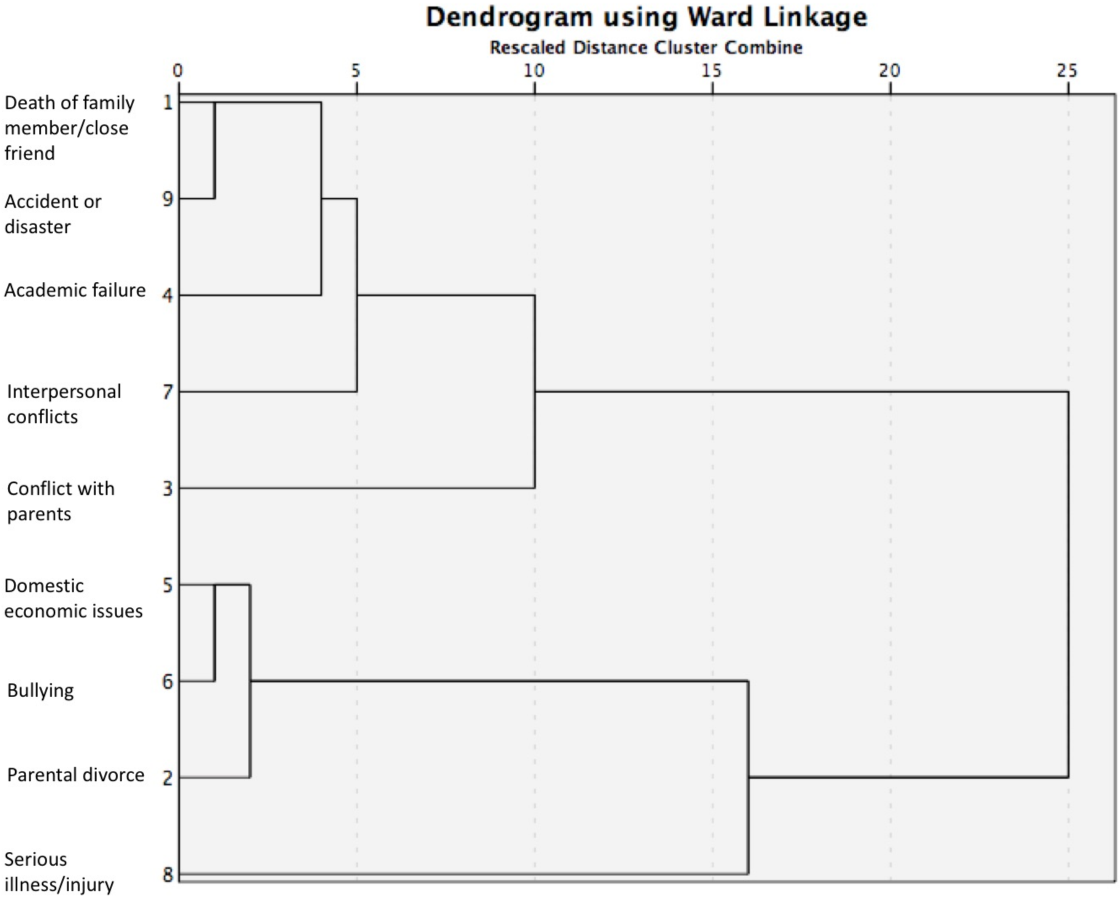

Note: group 2, n=519

Bootstrap analysis summary showing the indirect effects of past stress/trait-resilience on depression/current stress via PTG and PTSS.

Independent variables	Mediator variable	Dependent variable	*a* Path coefficient (A-B)	*b* Path coefficient (B-C)	Indirect effects and 95% CI [lower, upper]	c' coefficient (direct effect)
Past Stress	PTG	Depression	3.11***	-0.120***	-0.372 [-0.564, -0.200]	0.742***
	PTSS		6.44***	0.154***	0.999 [0.359, 0.881]	
	PTG	Current stress	3.11***	-0.007***	-0.023 [-0.035, -0.011]	0.241***
	PTSS		6.45***	0.014***	0.090 [0.067, 0.118]	
Trait-Resilience	PTG	Depression	1.46***	-0.005**	-0.001 [-0.011, -0.003]	-0.006 (n.s.)
	PTSS		-0.221*	0.018***	-0.004 [-0.007, -0.002]	
	PTG	Current stress	1.46***	-0.06***	-0.085 [-0.111, -0.060]	-0.311***
	PTSS		-0.221*	0.144***	-0.032 [-0.053, -0.011]	

Notes; PTG = Post Traumatic Growth, PTSS = Post Traumatic Stress Syndrome. A = Independent variable, B = Mediator variable, C = Dependent variable. *** $p< .0001$, ** $p< .001$ * $p< .005$
